# Overcoming T cell exhaustion and senescence in CAR T cell therapy for solid tumors

**DOI:** 10.1007/s12094-026-04383-9

**Published:** 2026-05-07

**Authors:** Ignacio Pérez-Criado, Mariona Figols, Ainhoa Moya-Sevilla, Ana Bautista, Daniela Gómez-Díaz, Albert Font, Juan Martin-Liberal, Vicenç Ruiz de Porras

**Affiliations:** 1 Medical Oncology Department, Althaia Xarxa Assistencial Universitària de Manresa, C/ Dr. Joan Soler, 1-3, 08243 Manresa, Spain; 2 GRET and Toxicology Unit, Department of Pharmacology, Toxicology and Therapeutic Chemistry, Faculty of Pharmacy and Food Sciences, University of Barcelona, Av. Joan XXIII, 27-31, 08028 Barcelona, Spain; 3 Medical Oncology Department, Catalan Institute of Oncology, Camí de les Escoles, s/n, 08916 Badalona, Spain; 4 CARE Program, Germans Trias i Pujol Research Institute (IGTP), Camí de les Escoles, s/n, 08916 Badalona, Spain; 5 Badalona Applied Research Group in Oncology (B·ARGO), Catalan Institute of Oncology, Camí de les Escoles, s/n, 08916 Badalona, Spain

**Keywords:** CAR T cell therapy, T cell exhaustion, Senescence, Solid tumors

## Abstract

Chimeric antigen receptor (CAR) T cell therapies have demonstrated remarkable efficacy in hematologic malignancies; however, their clinical benefit in solid tumors remains limited. A major barrier is T cell dysfunction, particularly exhaustion and senescence, which impair persistence, effector function, and durable tumor control. Targeting these dysfunctional states is therefore essential in order to improve CAR T cell efficacy in solid tumors.

This review summarizes recent preclinical strategies aimed at preventing or reversing CAR T cell exhaustion and senescence in solid malignancies. While both exhaustion and senescence are relevant dysfunctional states, the preclinical evidence summarized in this review is currently more extensive for modulation of exhaustion-associated programs than for direct reversal of canonical T cell senescence. Approaches are organized according to their primary mechanistic focus, including gene editing, metabolic modulation, receptor redesign, and remodeling of the tumor microenvironment.

Across these mechanistic categories, reported benefits include enhanced CAR T cell persistence, reduced expression of inhibitory receptors, such as PD-1, LAG-3, and TIM-3, preservation or restoration of memory-like phenotypes, and improved antitumor cytotoxicity. Notably, combinatorial strategies targeting multiple dysfunction pathways consistently demonstrate superior efficacy in preclinical models. Despite these advances, important translational challenges remain, including the limited predictive value of current preclinical systems, potential safety concerns, and the manufacturing complexity associated with increasingly engineered cell products.

Collectively, preclinical evidence supports the rational integration of complementary approaches to generate next-generation CAR T cells capable of resisting dysfunction and maintaining activity within immunosuppressive solid tumor microenvironments. Further validation in clinically relevant models will be critical to facilitate translation into safe and durable cancer immunotherapies.

## Introduction

Immunotherapy has emerged as one of the most promising strategies for the treatment of solid tumors by harnessing the patient’s immune system to combat malignant cells. Immune checkpoint inhibitors (ICIs), such as anti-Programmed death 1 (PD-1)/Programmed death ligand 1 (PD-L1) and anti-Cytotoxic T Lymphocyte Antigen 4 (CTLA-4) antibodies, currently represent the most widely used approach in clinical practice due to their ability to induce durable responses in a subset of patients [1, 2]. However, new therapeutic modalities have gained relevance in recent years, including cellular therapies, with chimeric antigen receptor (CAR) T cell therapy being an area of special interest. Among these approaches, interest has increasingly shifted toward understanding intrinsic T cell dysfunction—particularly exhaustion and senescence—which has emerged as a critical determinant of therapeutic efficacy in solid tumors.

CAR T cell therapy is an innovative strategy that involves the genetic modification of patient T cells to enable recognition and elimination of tumor cells. The basic CAR structure consists of an extracellular antigen-recognition domain, usually derived from an antibody fragment, a transmembrane region linking the extracellular and intracellular components, and an intracellular signaling domain that triggers T cell activation upon antigen engagement [3, 4]. Despite these sophisticated design features, CAR T cell activity is profoundly shaped by both tumor-intrinsic and host-related factors, many of which are uniquely challenging in solid tumors.

**Fig. 1:**
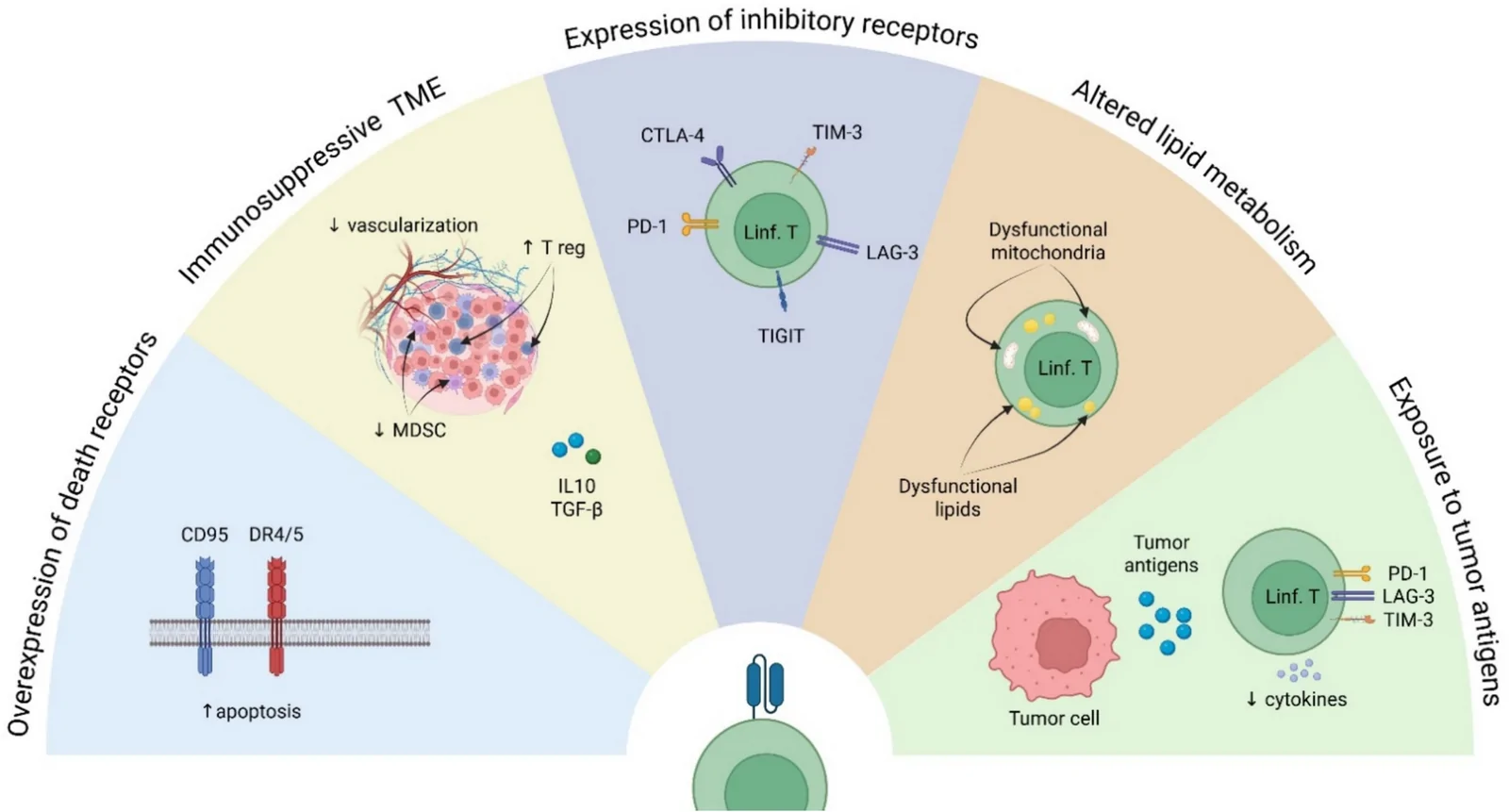
Cellular and molecular mechanisms involved in CAR T cell exhaustion and senescence in solid tumors. *CTLA-4* cytotoxic T lymphocyte antigen 4, *LAG-3* lymphocyte-activation gene 3, *MDSC* myeloid-derived suppressor cells, *PD-1* programmed death 1; programmed death ligand 1 (PD-L1), *TIM-3* T-cell immunoglobulin and mucin domain 3, *TIGIT* T-cell immunoreceptor with Ig and ITIM, *TME* tumor microenvironment. Created with BioRender.com

While CAR T cell therapy has transformed the management of hematologic malignancies, its effectiveness in solid tumors remains limited mainly due to the greater biological complexity of these tumors.[5]. Several factors contribute to this reduced efficacy (Fig. 1). A major challenge is the toxicity profile, as on-target, off-tumor effects may arise when the target antigen is shared with healthy tissues, leading to unintended immune-mediated damage [6]. Antigen heterogeneity further restricts CAR T cell activity, as variable antigen expression within the tumor limits complete eradication and promotes escape mechanisms such as antigen loss [7].

In addition to antigen-related barriers, the physical and structural characteristics of solid tumors present substantial obstacles. The dense extracellular matrix and complex stromal architecture hinder CAR T cell trafficking and reduce direct engagement with tumor cells, thereby limiting cytotoxic efficacy [8]. Moreover, even when CAR T cells reach their target, some solid tumors exhibit intrinsic resistance to CAR T-mediated killing, driven by factors, such as interferon gamma (IFNγ) receptor pathway deregulation, expression of anti-apoptotic molecules, and activation of immunosuppressive signaling networks [9]. Differences in CAR design can also influence therapeutic outcomes, with constructs incorporating costimulatory domains such as 4-1BB demonstrating improved persistence compared with other configurations [10]. Together, these extrinsic barriers delineate a highly restrictive environment that limits CAR T cell expansion, persistence, and effector function.

Superimposed on these external limitations are intrinsic barriers within the T cell compartment itself. T cell exhaustion and senescence profoundly impair therapeutic potency, arising in part from chronic antigen stimulation, which progressively diminishes effector functions, cytokine production (tumor necrosis factor alpha (TNFα), IFNγ and IL-2), and cytotoxic capacity while upregulating inhibitory receptors [11]. The immunosuppressive nature of the tumor microenvironment (TME) further exacerbates dysfunction through hypoxia, acidosis, nutrient deprivation, suppressive immune populations, and inhibitory cytokines, all of which impair T cell metabolism, proliferation, and survival [11, 12]. In addition, inhibitory receptors, such as PD-1, CTLA-4, Lymphocyte-activation gene 3 (LAG-3), T cell immunoglobulin and mucin domain 3 (TIM-3), and T cell immunoreceptor with Ig and ITIM (TIGIT), reinforce exhaustion pathways, while TME-driven metabolic alterations further compromise mitochondrial fitness and cellular function [13–15]. Consequently, intrinsic dysfunction of the T cell compartment has emerged as one of the most critical barriers to successful CAR T cell therapy in solid tumors, making the reversal or prevention of exhaustion and senescence a central goal in current research (Fig. 1).

Although T cell exhaustion and T cell senescence share some functional consequences, including impaired proliferation, reduced fitness, and diminished antitumor activity, they are not interchangeable biological states. T cell exhaustion is typically driven by persistent antigen exposure and chronic stimulatory signaling and is characterized by progressive loss of effector function, sustained expression of inhibitory receptors, such as PD-1, TIM-3, LAG-3, and TIGIT, and the establishment of a specific transcriptional and epigenetic program [16, 17]. By contrast, T cell senescence is more commonly associated with terminal differentiation and durable cell-cycle arrest, and has classically been linked to loss of CD27 and CD28, acquisition of markers, such as CD57 and Killer cell lectin-like receptor subfamily G member 1 (KLRG1), telomere attrition or dysfunction, DNA damage responses, and activation of cell cycle regulatory pathways including p16 and p21 [18]. While some degree of overlap may occur under conditions of chronic stimulation, repeated expansion, or tumor-induced stress, both states should not be considered equivalent. Importantly, the currently available preclinical evidence in CAR T cell therapy for solid tumors is substantially more robust for exhaustion-related phenotypes than for bona fide senescence reversal. Accordingly, this review addresses both concepts while acknowledging that most of the interventions identified to date primarily target exhaustion-like dysfunction [19, 20].

Given the multifactorial nature of these limitations, and considering that most currently available approaches predominantly target exhaustion-associated dysfunction, multiple innovative strategies are being explored. The strategies include genetic engineering to enhance metabolic fitness, pharmacologic interventions to reverse inhibitory signaling, modulation of the TME, and combinatorial approaches involving cytokines or checkpoint blockade (Table 1). Overcoming these barriers is essential to improve the effectiveness of CAR T cell therapy in solid tumors. Thus, the aim of this review is to provide a comprehensive synthesis of emerging experimental approaches designed to counteract T cell exhaustion and senescence in cellular therapies for solid malignancies. By critically evaluating preclinical strategies targeting metabolic pathways, transcriptional regulators, receptor engineering, and the TME, this review seeks to identify promising therapeutic avenues with potential for clinical translation.

**Table 1:** Comparative overview of mechanisms of action, experimental models, main dysfunction addressed, and functional outcomes across the analyzed strategies

Mechanism of action	Ref.	Intervention	Experimental model	Main dysfunction addressed	In vitro results	In vivo results
Metabolic modulation	Xu et al. [28]	Inhibition of cPLA2α with ATK in T-Lip/ATK cells	MC38 colorectal cancer and B16F1 melanoma cell lines + syngeneic immunocompetent MC38 mouse model + human ex vivo tissue	Both (predominantly senescence-related)	↓ ROS, apoptosis, lipid droplets accumulation, senescence-associated cytokines ↑ cytotoxicity	↓ Tumor growth, Ki67^⁺^ cells ↑ Apoptosis, IFNγ, TNFα, granzyme B, CD8^⁺^ infiltration
	Yan et al. [29]	LXRβ deletion in CAR T cells via CRISPR-Cas9	Syngeneic immunocompetent murine colorectal cancer (MC38-OVA) and melanoma (B16F10-OVA) models)	T cell Exhaustion	↓ Autophagy and apoptosis ↑ LXRβ-KO CAR T cells	↓ PD-1, LAG-3 ↑ Tumor regression, survival
Gene editing	Lei et al. [37]	BATF degradation via eMIATA	Orthotopic glioma murine model	T cell Exhaustion	↓ PD-1, LAG-3 ↑ IFNγ, IL-2, cytotoxicity	↓ Tumor size ↑ Tumor clearance, survival
	Zhang et al. [38]	BATF deletion in CAR T cells via CRISPR-Cas9	Mesothelioma and melanoma xenografts and PDX models	T cell Exhaustion	↓ PD-1, TIGIT, LAG-3 ↑ Cytotoxicity, IFNγ, granzyme B, IL-2, central memory phenotype	↓ Tumor growth ↑ CD8^⁺^ infiltration, CAR T persistence, central memory phenotype
	Kumar et al. [41]	Cbl-b deletion via CRISPR-Cas9	Syngeneic immunocompetent murine colorectal cancer (MC38-OVA) and melanoma (B16F10-OVA) models)	T cell Exhaustion	↓ PD-1, TIM-3, LAG-3 ↑ IFNγ, TNFα, IL-2, granzyme B	↓ Tumor growth, PD-1^⁺^TIM-3^⁺^ cells ↑ CD8^⁺^ infiltration, IFNγ, TNFα, granzyme B, sustained cytotoxicity, survival
	Prinzing et al. [44]	DNMT3A deletion in anti-CD19 CAR T- cells	Orthotopic glioma and osteosarcoma murine models	T cell Exhaustion	↓ Exhaustion-related genes, dysfunction-associated cytokines, PD-1, TIM-3, LAG-3 ↑ Effector genes, proliferation, memory phenotype	↓ Tumor growth ↑ Tumor clearance, persistence, central memory
	Schelker et al. [46]	Overexpression of LMO4 in CD8^⁺^ T cells	Murine syngeneic melanoma (B16_KVP_) and lung carcinoma (LLC1-OVA) models	Senescence/stemness-memory preservation	↓ Terminal effectors ↑ proliferation and persistence, stem-like phenotype, IL-2, IFNγ, TNFα, memory-associated genes	↑ Expansion and accumulation, tumor control and survival (B16KVP), tumor control (LLC1-OVA)
CAR structural redesign	Zhang et al. [48]	CAR redesign using an rTCR-CAR architecture targeting EDB-FN	A549 and U87MG tumor-bearing NCG mouse models	T cell Exhaustion	↓ PD-1, TIM-3, LAG-3 ↑ Cytotoxicity	↓ Tumor growth, tumor vasculature ↑ CD8^⁺^ infiltration, rTCR-CAR proliferation, *naïve-like* phenotype
	Liang et al. [49]	SMAD7 co-expression in anti-HER2 CAR T-cell	Xenograft murine models (HER2 + Fluc + HeLa cells) and HER2 + tumor organoids	T cell Exhaustion	↓ PD-1, LAG-3, TIGIT, IL-6, IL-8, TNFα, apoptosis ↑ Cytotoxicity, memory phenotype, effector phenotype	↓ Serum inflammatory cytokines ↑ Survival, infiltration, memory phenotype
Microenvironment modulation	Gao et al. [53]	S1PR3 inhibition with TY-52156	Syngeneic immunocompetent murine colorectal cancer (MC38) and breast cancer (4T1) models	T cell Exhaustion	↓ IL-6, TGF-β, PD-1, TIM-3, LAG-3 ↑ IFNγ, IL-2, TNFα, cytotoxicity, memory phenotype	↓ Tumor growth, PD-1, TIM-3, LAG-3 ↑ Survival, complete responses, CAR T-cell infiltration, IFNγ, IL-2, granzyme B
	Nguyen et al. [55]	HDACi (MS-275) combined with ACT	Syngeneic immunocompetent murine tumor models (BALB/c CMS-5 and C57BL/6 B16-F10)	T cell Exhaustion	–	↓ Tumor load–induced exhaustion ↑ Tumor regression, survival, CD8^⁺^ proliferation, granzyme B, IFNγ
	Zhao et al. [58]	Constitutive IL-10 expression in CAR T cells	Syngeneic and xenograft murine tumor models (colon, melanoma, breast, and pancreatic cancer)	T cell Exhaustion	↓ PD-1, TIM-3, exhaustion ↑ Proliferation and expansion, IL-2, IFNγ, TNFα, granzyme B, maintained cytotoxicity	↓ Serum inflammatory cytokines ↑ complete tumor regression, CAR persistence, memory phenotype, overall survival

*ACT* adoptive cell therapy, *ATK* arachidonyl trifluoromethyl ketone, *BATF* basic leucine zipper ATF-like transcription factor, *cPLA2α* cytosolic phospholipase A2 alpha, *eMIATA* endosome-microautophagy targeting chimera, *HDACi *class I histone deacetylase inhibitors, *IFNγ* interferon gamma, *IL* interleukin, *LAG-3* lymphocyte-activation gene 3, *LMO4* lIM-domain-only 4, *LXRβ* liver X receptor beta, *OVA* ovalbumin, *PD-1* programmed cell death protein 1, *ROS* reactive oxygen species, *TCR* T-cell receptor, *TGF-β* transforming growth factor beta, *TIGIT* T-cell immunoreceptor with Ig and ITIM, *TIM-3* T-cell immunoglobulin and mucin domain 3, *TNFα* tumor necrosis factor alpha, *SMAD7 *SMAD family member 7

To support this objective, a focused literature search was conducted in PubMed using relevant MeSH terms related to “T cell exhaustion”, “senescence”, “CAR T cell therapy”, and “solid tumors”. The search was restricted to high-impact studies published between 2015 and 2025. Priority was given to original research reporting experimental in vivo data and describing therapeutic or mechanistic interventions in CAR T cell platforms for solid tumors. However, a limited number of studies based on closely related adoptive or engineered T cell systems were also included when they provided particularly relevant mechanistic insight into exhaustion- or senescence-associated dysfunction with clear translational relevance for CAR T cell therapy. Review articles, commentaries, and studies lacking primary data were excluded. This approach was intended to provide a representative and up-to-date narrative synthesis rather than a formal systematic review.

## Therapeutic strategies to mitigate T cell exhaustion and senescence

### Metabolic modulation to reverse cellular exhaustion

Alterations in lipid metabolism and cholesterol deficiency are key mechanisms underlying T cell exhaustion in solid tumors. Activation of the MAPK and STAT1/3 pathways increases levels of cytosolic phospholipase A2 alpha (cPLA2α), promoting the accumulation of free fatty acids, the formation of lipid droplets, and, ultimately, cellular exhaustion [13, 21–23]. Activated T cells require cholesterol for expansion, yet within the TME they experience a deficiency of this lipid, in contrast to its abundance in tumor cells and MDSCs. This dysregulation suppresses cholesterol uptake, inhibits mTORC1 signaling, and promotes dysfunction of CD8 + T cells [24–27].

**Fig. 2:**
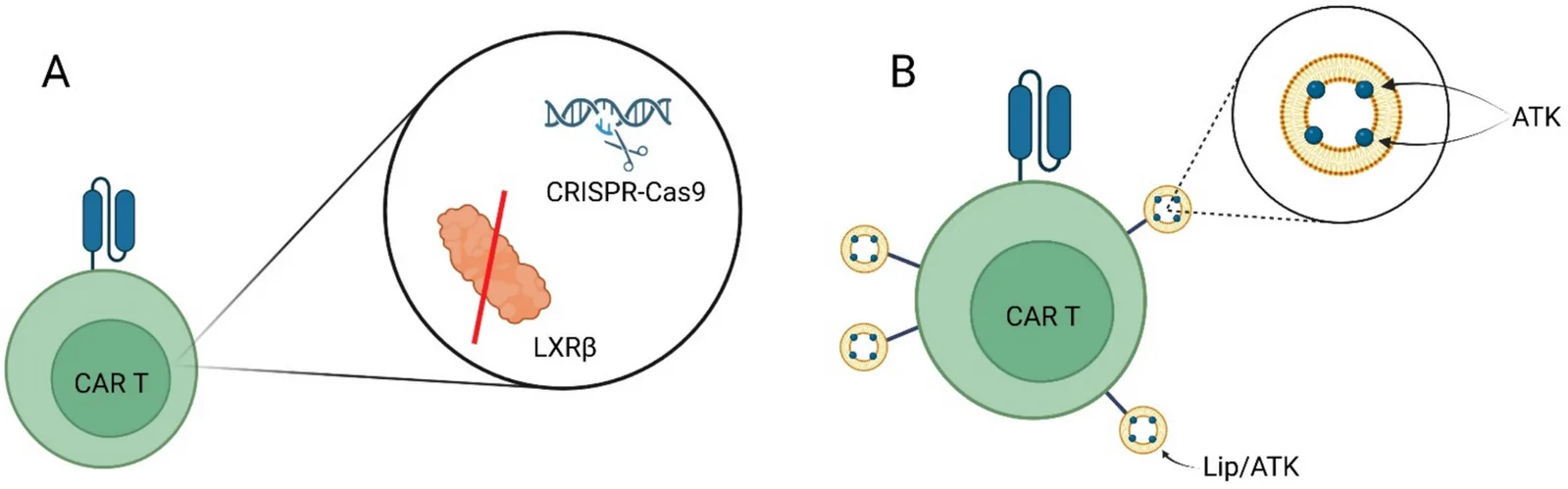
Metabolic modulation strategies to enhance CAR T cell functionality. **A** CRISPR-Cas9-mediated deletion of Liver X receptor beta (LXRβ) promotes intracellular cholesterol accumulation, thereby enhancing T cell effector function. **B** T cell-anchored liposomes (T-Lip/ATK) deliver the cytosolic phospholipase A2 alpha (cPLA2α) inhibitor ATK via liposome anchoring, mitigating lipid-induced T cell dysfunction. Created with BioRender.com

Strategies aimed at reprogramming lipid and cholesterol metabolism have demonstrated efficacy in preserving CAR T cell functionality. In this context, pharmacologic inhibition of cPLA2α using T cell-anchored liposomes (T-Lip/ATK) reduced in vitro cPLA2α levels, reactive oxygen species, early apoptosis, and lipid-droplet accumulation, while increasing cytotoxicity and restoring CD27/CD28 expression. It also decreased the production of proinflammatory cytokines associated with senescence (IL-1β, IL-6, IL-10, and TGF-β). In vivo, in colorectal cancer models, the combination of T-Lip/ATK with anti-PD-L1 reduced tumor growth, decreased the proportion of Ki67 + tumor cells, and increased apoptosis, CD8 + T cell infiltration, and production of IFNγ (up to 1.5-fold), TNFα and granzyme B. In B16F1 melanoma models, the combination with anti-PD-L1 reproduced these effects although T-Lip/ATK monotherapy was less effective due to the highly immunosuppressive nature of the tumor [28] (Fig. 2).

Genetic editing of the Liver X receptor beta (LXRβ) through CRISPR–Cas9 (LXRβ-KO) in CAR T cells increased free cholesterol accumulation, restored mTORC1 signaling, enhanced the expression of genes related to lipid metabolism and glycolysis, and reduced autophagy and apoptosis. It also improved viability and proliferation after repeated antigen stimulation. In vivo, in lung carcinoma (LLC-OVA) and melanoma (B16F10-OVA) models, greater tumor regression was observed, along with increased CAR T cell infiltration, reduced expression of exhaustion markers (PD-1 and LAG3), and improved survival [29] (Fig. 2; Table 1).

### Genetic editing to modulate transcription factors and epigenetic regulators

Post-translational and epigenetic modifications play a decisive role in T cell dysfunction in solid tumors. Targeting transcription factors and epigenetic regulators has emerged as a strategy to enhance CAR T cell persistence and anti-tumor activity. Basic Leucine Zipper ATF-Like Transcription Factor (BATF), a key regulator of T helper cell differentiation that has also been associated with T cell exhaustion [30–35], has been targeted through two main approaches: protein degradation via the endosome-microautophagy targeting chimera (eMIATAC) system and gene editing using CRISPR-Cas9. The eMIATAC system, designed to overcome the specificity limitations of traditional methods, such as RNA interference and CRISPR/Cas9 [36], increased in vitro production of effector cytokines (IL-2 and IFNγ), reduced exhaustion markers (PD-1 and LAG-3), and enhanced cytotoxicity and the speed of tumor cell lysis. In vivo, in an orthotopic glioma model, it enhanced tumor-cell elimination, significantly reduced tumor size, and improved survival [37].

**Fig. 3:**
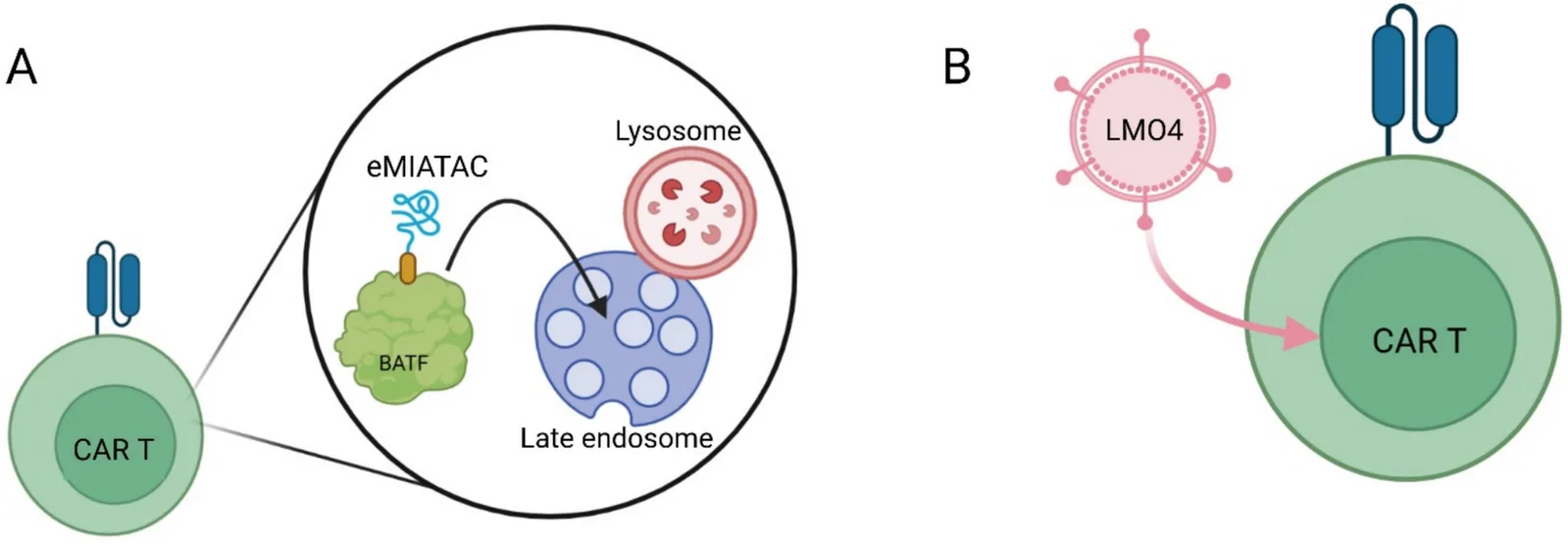
Genetic editing strategies to modulate transcriptional and epigenetic regulators in CAR T cells. **A** Targeted degradation of Basic leucine zipper ATF-Like transcription factor (BATF) using the endosome-microautophagy targeting chimera (eMIATAC) system enhances CAR T cell effector function and limits exhaustion through lysosomal targeting. **B** LIM-domain-only 4 (LMO4) overexpression promotes stem-like memory phenotypes by enhancing IL-21-STAT3 signaling. Created with BioRender.com

Genetic depletion of *BATF* (*BATF*-KO) in anti-mesothelin CAR T cells increased in vitro cytotoxicity and cytokine production, as well as the proportion of CAR T cells with a central memory phenotype (CD45RA–CD62L +). It also induced a transcriptomic profile with higher expression of genes associated with effector function and proliferation (*PRF1, GZMB, IFNG* and *IL2RB*) and lower expression of exhaustion-associated genes (*PDCD1, TOX, TIGIT* and *LAG3*). In vivo, in NCI-H226–derived xenografts and patient-derived xenografts (PDX) of pancreatic adenocarcinoma, *BATF*-KO CAR T cells reduced tumor growth, increased intratumoral CD8 + infiltration, increased the proportion of CAR T cells with a CD45RO + CCR7 + phenotype in blood and tumor, improved persistence in peripheral blood, and completely eradicated 80% of tumors while maintaining protection after tumor rechallenge, without significant toxicity [38] (Fig. 3).

Casitas B-lineage lymphoma-b (Cbl-b), a ubiquitin ligase that acts as a negative regulator of T cell activation [39, 40], was eliminated using CRISPR-Cas9. In tumor-infiltrating T lymphocytes, this increased in vitro IFNγ, TNFα, granzyme B and IL-2, while reducing the expression of inhibitory receptors (PD-1, TIM-3, and LAG-3). In vivo, in anti-HER2 CAR T cells and MC38 colon adenocarcinoma and B16-F10 melanoma models, Cbl-b deletion resulted in reduced tumor burden, lower proportions of PD-1 + TIM-3 + cells, more durable cytotoxicity, increased cytokine production, and significantly improved survival, without evidence of significant toxicity [41].

DNMT3A, a DNA methyltransferase implicated in methylation changes associated with T cell dysfunction [42, 43], was suppressed in CAR T cells using CRISPR-Cas9. In vitro, DNMT3A deletion increased the expression of effector genes (*GZMB, PRF1* and *IFNG*), reduced expression of exhaustion-related genes (*PDCD1, TOX* and *NR4A1*), decreased inflammatory cytokines (IL-10, IL-13 and IL-6), and increased the proportion of memory-associated phenotypes (*TCF7, LEF1, IL7R, CD62L* and *CD27*). In vivo, in osteosarcoma and orthotopic glioma models, it increased tumor control (complete elimination in 50% of tumors), as well as persistence in bone marrow and blood, the proportion of CAR T cells with a central memory phenotype (CD45RA–CD62L +), and IFNγ and TNFα production [44].

Finally, LIM-domain-only 4 (LMO4), a transcriptional regulator that enhances T cell memory phenotypes [45], was overexpressed in CD8 + T cells specific for tumor antigens. In in vitro, it increased the proportion of stem-like cells (CD62L⁺KLRG1⁻), simultaneous cytokine production (IL-2, IFNγ and TNFα), and the expression of memory and persistence genes (*TCF7* and *SOCS3*). In in vivo, in murine models of lung carcinoma (LLC1-OVA) and melanoma (B16KVP), it increased T cell expansion and accumulation in spleen, lymph nodes, and lungs, promoted long-term memory persistence, and improved tumor control and survival in melanoma [46] (Fig. 3; Table 1).

### Structural redesign of CAR/ T cell receptors (TCR)

**Fig. 4:**
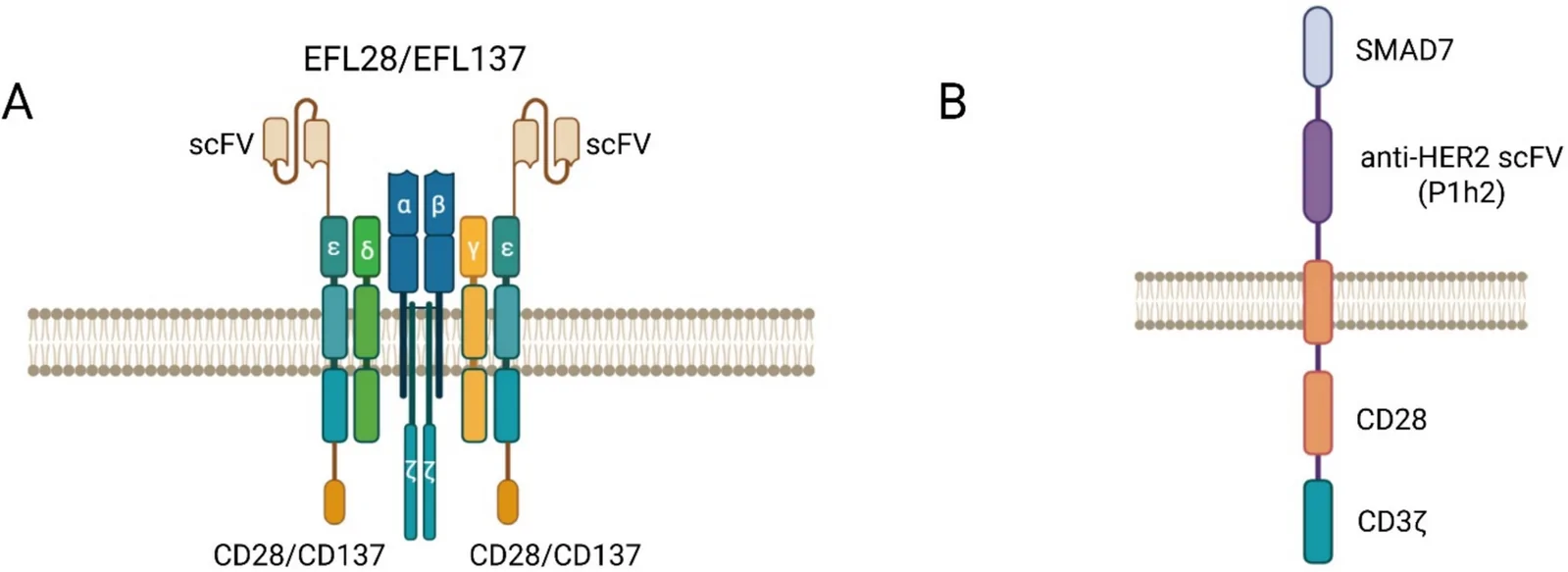
Structural redesign of CAR/T cell receptors (TCR) to overcome T cell exhaustion. **A** rTCR-CAR constructs (EFL28/EFL137) targeting the fibronectin containing the extra domain B (EDB-FN) isoform integrate TCR and CAR signaling modules, optimizing immunological synapse formation and enhancing effector function. **B** Co-expression of SMAD7 in anti-HER2 CAR T cells blocks TGF-β-mediated immunosuppression, promoting memory differentiation and reducing the expression of exhaustion markers. Created with BioRender.com

Engineering costimulatory domains and incorporation of TCR components have emerged as a strategy to optimize the immunological synapse formation and persistence in solid tumors. A notable example is the development of redirected TCR-CAR receptors targeting fibronectin containing the extra domain B (EDB-FN), an oncofetal isoform expressed exclusively in tumor neovasculature and certain malignant cells, absent in normal adult tissues [47]. Although second-generation CAR T cells have shown limited efficacy against this target due to difficulties in TME infiltration, fusion of an anti-EDB-FN scFv to engineered TCR complexes incorporating costimulatory domains—CD28 (EFL28) or 4—1BB (EFL137)—improved synapse organization. In in vitro, rTCR-CAR EFL28 cells exhibited cytotoxic activity comparable to that of conventional CAR T cells, while displaying higher expression of CD69, CD25, CD107a, and granzyme B, and outperforming EFL137 in cytotoxic potency. In vivo, in A549 and U87MG models, EFL28 cells reduced tumor growth, selectively decreased tumor vascular density without affecting healthy tissues, enhanced T cell proliferation and infiltration, and attenuated exhaustion marker expression (PD-1, TIM-3, and LAG-3) [48] (Fig. 4).

Co-expression of SMAD family member 7 (SMAD7), a negative regulator of the TGF-β pathway, in anti-HER2 CAR T cells bearing a CD28–CD3ζ signaling domain (S7H28z) was developed to counteract TGF-β-mediated immunosuppression. This CAR design increased cytotoxicity in the presence of TGF-β, enhanced proliferation and survival after repeated antigenic stimulation, reduced inhibitory receptor expression (PD-1, LAG-3, and TIGIT), and increased the proportion of memory phenotypes (higher expression of CD27, TCF1, Jun, KLF9). In in vivo, in HeLa HER2 + xenografts, it improved survival, reduced serum inflammatory cytokines, and increased persistence of effector phenotypes (day + 14) and memory phenotypes (day + 28). In HER2 + ovarian cancer organoids, it increased tumor infiltration, malignant cell apoptosis and the proportion of central memory T cells (CD45RA–CD62L +), while reducing PD-1, TIM-3, LAG-3, and inflammatory cytokines (IL-6 and IL-8) [49] (Fig. 4**; **Table 1).

### Strategies directed at the TME

**Fig. 5:**
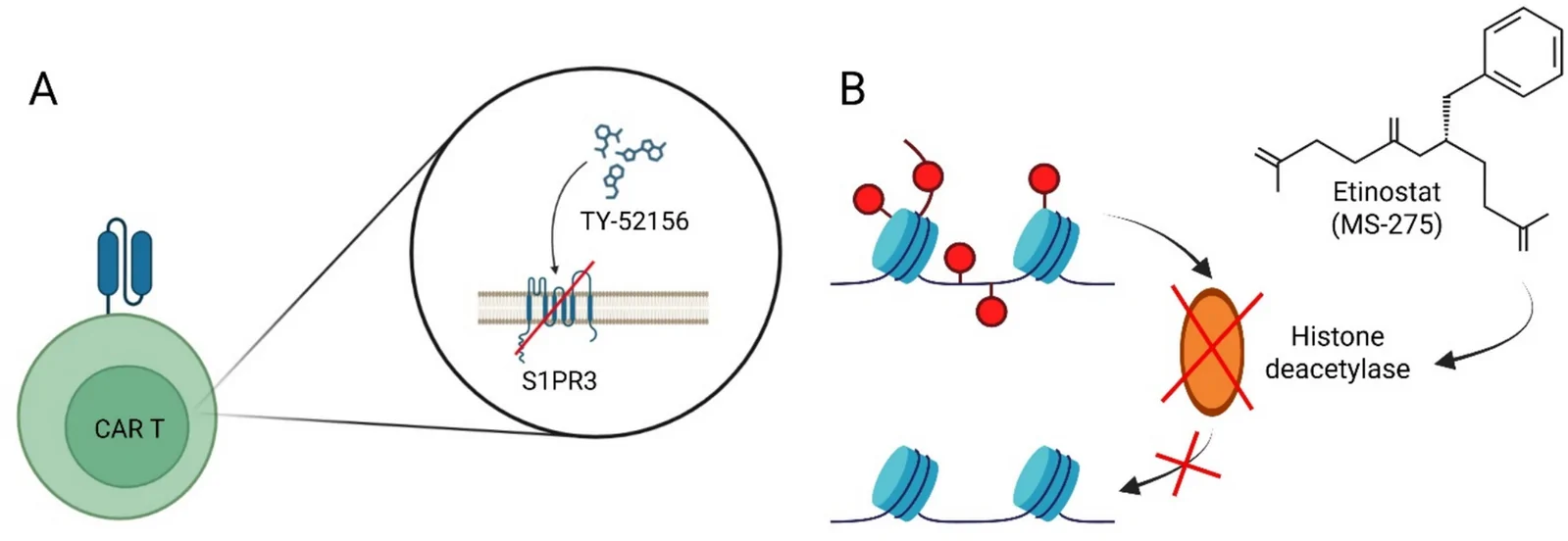
Tumor microenvironment-targeting strategies to enhance CAR T-cell function. **A** Pharmacological inhibition of the Sphingosine-1-phosphate receptor (S1PR3) with TY-52156 attenuates tumor microenvironment-driven exhaustion signals in CAR T cells. **B** Treatment with the histone deacetylase inhibitor entinostat (MS-275) promotes chromatin remodeling, thereby enhancing effector function and memory differentiation in adoptively transferred T cells. Created with BioRender.com

The TME is a critical barrier to CAR T cell efficacy in solid tumors. It is characterized by hypoxia, acidosis, nutrient scarcity, and the presence of immunosuppressive cells, making it one of the most intensively studied therapeutic targets. Sphingosine-1-phosphate (S1P) is a bioactive lipid molecule that binds to its receptor Sphingosine-1-phosphate receptor 3 (S1PR3), promoting tumor development through activation of PI3K, MAPK or PLC pathways, modulating macrophage phenotypes toward immunosuppressive profiles, and regulating anti-inflammatory cytokines within the TME [50–52]. The use of the selective S1P3 inhibitor TY-52156 in combination with CAR T cells in murine models of melanoma (B16-Ovalbumin (OVA)) and lung carcinoma (LLC-OVA) increased IFNγ, TNFα, and IL-2 production, enhanced cytotoxic function, and expanded the proportion of memory-like T cells (CD44⁺CD62L⁺). This combination also reduced the expression of exhaustion markers (PD-1, TIM-3, and LAG-3) and induced a transcriptional program associated with attenuated T cell exhaustion. In vivo, the combined treatment promoted tumor regression and achieved near-complete response rates, increased CAR T cell infiltration, decreased PD-1, TIM-3, and LAG-3 expression, and improved survival, without evidence of significant toxicity [53] (Fig. 5; Table 1).

High tumor burden is known to induce TME remodeling that drives profound T-cell dysfunction [54]. In this context, class I histone deacetylase inhibitors (HDACi), such as MS-275 (entinostat), reprogram aberrant chromatin regulation, sensitizing tumors, and enhancing immune responses [55]. When combined with T-cell therapy, MS-275 induced complete tumor regression by day 14 in fibrosarcoma (CMS5) models, improved survival, and increased CD8⁺ T-cell proliferation and persistence. Treatment reversed immunosuppressive gene signatures, enhanced granzyme B production and cytotoxicity, and promoted a terminal effector phenotype (TIM-3^hi CD44⁺ CD62L⁻ TCF1⁻ CD127⁻ KLRG1⁺). In in vivo, in colon adenocarcinoma (MC38-gp33) models, MS-275 further augmented tumor regression and survival, increased IFNγ, granzyme B, and CD107a production, and skewed T cells toward an effector phenotype with a reduced proportion of progenitor-exhausted cells [56] (Fig. 5; Table 1).

Finally, interleukin 10 (IL-10), traditionally associated with immunosuppression, has been shown to restore mitochondrial metabolism and CD8 + T-cell function within tumor environments when administered as the fusion protein IL-10-Fc [57]. A redesigned CAR T-cell platform enabling constitutive IL-10 expression (IL-10 CAR) increased in vitro proliferation and expansion after repeated stimulation, increased IL-2, IFNγ, TNFα and granzyme B production, and reduced PD-1 and TIM-3 expression. It also maintained cytotoxicity under repeated antigen stimulation and exhibited a transcriptomic profile associated with higher effector function and reduced exhaustion. In in vivo, across models of colon, melanoma, breast and pancreatic cancer, IL-10 CAR T cells increased complete tumor regression, expansion and persistence in blood and bone marrow, and the proportion of memory phenotypes, along with reduced serum inflammatory cytokines, ultimately improving survival [58] (Table 1).

## Discussion

Functional T cell exhaustion represents a major barrier to effective cellular therapy in solid tumors [59]. In contrast to hematologic malignancies, where CAR T cell therapies have delivered substantial clinical benefit and are already a standard therapeutic option, activity in solid tumors has been limited by a progressive loss of functionality, persistence, and cytotoxic capacity [5]. In this context, identifying strategies that prevent, delay, or reverse exhaustion and senescence programs is essential to optimize the therapeutic performance of cellular therapies in solid tumors.

This review encompassed twelve preclinical studies evaluating innovative strategies to mitigate T cell dysfunction in solid tumor models. Although most were conducted in CAR T cell settings, a limited number were derived from closely related adoptive or engineered T cell platforms and were included because of their mechanistic relevance to dysfunctional T cell states in solid tumors. Overall, the strongest evidence related to exhaustion-like phenotypes, whereas direct evidence for bona fide senescence reversal remained comparatively limited. Interventions were categorized into four mechanistic groups: (i) gene editing (five studies), (ii) metabolic modulation (two studies), (iii) receptor structural redesign (two studies), and (iv) modulation of the tumor microenvironment (three studies). Across studies, enhanced cytotoxic function of engineered T cells was consistently reported, while reductions in exhaustion marker expression (PD-1, TIM-3, and LAG-3), improved in vivo persistence, and survival benefits were observed in a substantial subset of models. Notably, approaches, such as S1PR3 inhibition with TY-52156, SMAD7 co-expression, and LXRβ suppression demonstrated particularly robust antitumor activity, including high rates of complete responses in selected animal models.

Despite methodological diversity, several convergent features emerged among the most successful approaches: preservation of memory phenotypes, reduced expression of inhibitory receptors, sustained cytotoxic capacity, and metabolic reprogramming toward more functional states. Together, these observations reinforce the view that T cell exhaustion is multifactorial and is best addressed through integrated, multi-pronged strategies. Notably, several studies evaluated interventions under sustained antigen exposure and/or immunosuppressive conditions designed to recapitulate key features of the TME, suggesting that at least some approaches can preserve T cell function even in hostile settings.

These findings should be interpreted in light of important limitations. All included studies were preclinical and employed heterogeneous experimental systems (murine models, in vitro assays, and organoid-based platforms), limiting direct clinical generalizability. In addition, the search was restricted to PubMed, which may under-capture negative or neutral studies. Nevertheless, the results are promising and broadly consistent across diverse strategies. Further validation in more translationally relevant models and, ultimately, in clinical trials will be required.

Beyond these general limitations, the translational feasibility of the reviewed strategies is likely to differ substantially. Pharmacologic or modular approaches, particularly those that could be combined with existing CAR T cell platforms without requiring extensive redesign of the cellular product, may represent comparatively realistic paths toward clinical implementation. By contrast, more heavily engineered strategies involving multiplex gene editing, complex receptor redesign, or constitutive expression of additional transgenes remain highly attractive conceptually, but may face substantial challenges related to manufacturing complexity, scalability, cost, product consistency, and regulatory approval. In addition, interventions that enhance T cell activity too broadly may raise safety concerns, including excessive immune activation, off-tumor effects, or altered long-term behavior of the infused cells. Taken together, these considerations underscore that preclinical efficacy alone should not be interpreted as a direct proxy for clinical feasibility, and that future work should prioritize not only biological activity but also safety, manufacturability, and translational practicality.

Despite these advances, several important gaps remain. First, the field still lacks clinically validated biomarkers that can reliably distinguish or quantify senescence-related dysfunction in CAR T cell settings, particularly in ways that are clearly separable from exhaustion-associated programs. Second, more predictive preclinical models are needed to better capture the cellular, stromal, and immunologic complexity of human solid tumors. Finally, the absence of standardized frameworks to compare exhaustion-rescue strategies across studies—including differences in model systems, phenotypic readouts, and functional endpoints—continues to limit cross-study interpretation and prioritization of the most promising approaches for clinical translation.

## Conclusions and future perspectives

Importantly, the findings summarized here have relevant implications for clinical translation and future research. Several identified approaches—particularly those leveraging molecules already used in other contexts or those that do not require complex manipulations of CAR T cells (e.g., MS-275, LXRβ suppression, or SMAD7 co-expression)—could be evaluated in early-phase clinical trials. By contrast, highly engineered cellular products involving multiple genetic modifications, constitutive transgene expression, or complex receptor redesign remain conceptually compelling but may require more extensive preclinical development before clinical translation. Other strategies that are more complex from a technical or regulatory standpoint will require further development in preclinical platforms before being considered for human studies. It will also be essential to advance experimental models that replicate as faithfully as possible human immune physiology and the TME. Ultimately, the rational integration of these strategies, whether individually or in combination, may contribute to the development of cellular therapies that are more effective, resistant to exhaustion, and better adapted to the complexity of solid tumors.

## Data Availability

Not applicable.

## Abbreviations

**ACT**: Adoptive cell therapy
**ATK**: Arachidonyl trifluoromethyl ketone
**BATF**: Basic leucine zipper ATF-like transcription factor
**CAR**: Chimeric antigen receptor
**Cbl-b**: Casitas B-lineage lymphoma-b
**CD**: Cluster of differentiation
**cPLA2α**: Cytosolic phospholipase A2 alpha
**CTLA-4**: Cytotoxic T-lymphocyte antigen 4
**DNMT3A**: DNA methyltransferase 3 alpha
**ECM**: Extracellular matrix
**EDB-FN**: Extra domain B-containing fibronectin
**eMIATAC**: Endosome-microautophagy targeting chimera
**HDACi**: Class I histone deacetylase inhibitors
**HER2**: Human epidermal growth factor receptor 2
**IFNγ**: Interferon gamma
**IL**: Interleukin
**KLRG1**: Killer cell lectin-like receptor subfamily G member 1
**LAG-3**: Lymphocyte-activation gene 3
**LMO4**: LIM-domain-only 4
**LXRβ**: Liver X receptor beta
**MDSC**: Myeloid-derived suppressor cell
**NOS2**: Nitric oxide synthase 2
**OVA**: Ovalbumin
**PD-1**: Programmed cell death protein 1
**PD-L1**: Programmed death-ligand 1
**PDX**: Patient-derived xenograft
**ROS**: Reactive oxygen species
**S1P**: Sphingosine-1-phosphate
**S1PR3**: Sphingosine-1-phosphate receptor 3
**scFv**: Single-chain variable fragment
**SMAD7**: SMAD family member 7
**TCR**: T-cell receptor
**TGF-β**: Transforming growth factor beta
**TIGIT**: T-cell immunoreceptor with Ig and ITIM
**TIM-3**: T-cell immunoglobulin and mucin domain 3
**TME**: Tumor microenvironment
**TNFα**: Tumor necrosis factor alpha
